# The complex aerodynamic footprint of desert locusts revealed by large-volume tomographic particle image velocimetry

**DOI:** 10.1098/rsif.2015.0119

**Published:** 2015-07-06

**Authors:** Per Henningsson, Dirk Michaelis, Toshiyuki Nakata, Daniel Schanz, Reinhard Geisler, Andreas Schröder, Richard J. Bomphrey

**Affiliations:** 1 Department of Biology, Lund University, Lund, Sweden; 2 LaVision GmbH, Göttingen, Germany; 3 Structure and Motion Laboratory, Royal Veterinary College, University of London, London, UK; 4 German Aerospace Centre (DLR), Göttingen, Germany

**Keywords:** aerodynamics, tomographic particle image velocimetry, desert locust, animal flight, wake deformation, span efficiency

## Abstract

Particle image velocimetry has been the preferred experimental technique with which to study the aerodynamics of animal flight for over a decade. In that time, hardware has become more accessible and the software has progressed from the acquisition of planes through the flow field to the reconstruction of small volumetric measurements. Until now, it has not been possible to capture large volumes that incorporate the full wavelength of the aerodynamic track left behind during a complete wingbeat cycle. Here, we use a unique apparatus to acquire the first instantaneous wake volume of a flying animal's entire wingbeat. We confirm the presence of wake deformation behind desert locusts and quantify the effect of that deformation on estimates of aerodynamic force and the efficiency of lift generation. We present previously undescribed vortex wake phenomena, including entrainment around the wing-tip vortices of a set of secondary vortices borne of Kelvin–Helmholtz instability in the shear layer behind the flapping wings.

## 1. Introduction

In order to understand the mechanics by which animals fly, it is important to understand their interaction with the air. This interaction, mediated by the flapping wings, leaves behind a wake or aerodynamic footprint that can be captured, quantified and subsequently assessed within the framework of aerodynamic theory. The utility of animal flight investigations using a quantitative flow imaging technique known as particle image velocimetry (PIV) has increased in recent times, mirroring advances in the technology available to researchers. The first experiments were limited to two-dimensional measurement slices through the wake, and could only calculate two-component velocity vectors within that plane. Laser power restricted the experiments to small measurement areas and low acquisition rates, typically lower than the wingbeat frequency of the animals flying in the wind tunnel [1,2]. The step to time-resolved data, where sampling rate is in excess of the wingbeat frequency, came some time later [3–5]. These studies marked the introduction to animal flight experiments of stereo-PIV, where three-component vectors could be arranged on the illuminated measurement plane through the wake. Until then, out-of-plane velocities had to be inferred from the flow structure and conservation of mass, or data drop-out due to particles leaving the illumination plane [2]. In studies of animal wakes, it has become commonplace to reconstruct wakes into a pseudo-three-dimensional arrangement by assembling, or stacking, stereo planes [3–5].

PIV measurements and processing can now be fully three-dimensional [6]. This new generation of techniques comes with the appealing prospect of capturing flow volumes. Time-resolved applications followed soon after [7]. The significant advantage of capturing wakes instantaneously is to dispense with the requirement of reconstructing pseudo-volumes in order to calculate flow derivatives in other dimensions, such as vorticity around axes other than that which is normal to the measurement plane. To date, the only tomographic (tomo) PIV study of animal flight is a time-resolved (1 kHz) study of desert locusts (*Schistocerca gregaria,* Forsskål) [8]. This study was limited because insufficient laser power restricted the maximum volume size to be thin relative to the gross wake geometry (*ca* 4 mm). The volumes were therefore capable of three-dimensional derivative calculations but too small to capture an entire wavelength of the wake. Ultimately, these mini-volumes had to be stacked to present an estimate of how the wake flow would appear in its entirety. In addition to highlighting drawbacks with stacking stereo planes (such as spatio-temporal resolution) that can be deduced from first principles [9], the first application of tomo-PIV to animal flight showed that stereo plane stacking is prone to systematic error due to wake deformation and made predictions of biases that may be introduced by this method [8]. Here we apply a state-of-the-art, large-volume, tomo-PIV apparatus to the same locust paradigm, testing those predictions exploring the detail of the wake structure that can be seen only when the wake is captured in its entirety, instantaneously.

The principal objectives for our experiments were twofold. First, we aimed to make the first recordings that captured the whole wavelength of the wake in one instant, presenting the most detailed three-dimensional quantitative flow visualizations of the wake of a flying animal to date. Second, we aimed to use our data to test our previously published hypotheses regarding wake development [8], and to measure how this wake development affects estimates of aerodynamic force and span efficiency [5,10–14]. A recent study on the wake development behind two fixed aerofoils that capture the key features of animal wakes—wing-tip and root vortices—shows that large-scale wake deformation takes place even over relatively short distances downstream of the wings [14]. In the light of that study, we sought to investigate deformation of the wake behind a live animal.

## 2. Material and methods

### 2.1. Animals and experimental procedure

We performed experiments on tethered desert locusts flying in an open circuit wind tunnel with a 1 m wide test section at the German Aerospace Centre (DLR) in Göttingen, Germany. The reasons for choosing desert locusts were because of their long-distance flight capabilities, that they are known to fly well whilst tethered, and that they are the most comprehensively described species of insect for kinematics [15], rigid body dynamics [16] and aerodynamics [17]. The aerodynamics of locusts have been studied in progressively greater detail over the years using smoke visualization [18], longitudinal PIV [19], transverse PIV [5] and time-resolved, small-volume, tomo-PIV [8], all of which provide a solid framework upon which the results from this large-volume, tomo-PIV experiment can build.

We recorded data from five individuals (Livefoods UK). The locusts were fixed at the exoskeleton on the ventral side of the thorax to a rigid tether (2.5 mm diameter) using cyanoacrylate glue. The tether was then mounted on a profiled sting and placed in the free stream with the hindwing trailing edge 10 mm in front of the illuminated volume. This is the closest possible position without the tip of the abdomen entering the volume. The sting was adjusted in pitch to give a body angle of 8°, which has been shown to be close to the pitch equilibrium angle [20], and flown at 3.3 ms^−1^, which is their equilibrium flight speed [20]. The ambient temperature was 26°C. Prior to data acquisition, each individual was allowed to fly on the tether for a few minutes to acclimatize; recordings commenced only when the locusts had adopted the full flight posture as defined by Weis-Fogh [21]. For each individual, 230 frames were recorded which corresponded to either 23 or 46 s of flight depending on our acquisition rate for that sequence: both 10 and 5 Hz sampling rates were used (see below).

Locusts tend to phase-lock to strobe light [22], particularly if the pulse frequency is close to their natural wingbeat frequency of approximately 20 Hz, or a factor of this. Since the laser used for this experiment was capable of repetition rates of 10 and 5 Hz, some of the locusts would phase-lock a few seconds after the laser had started flashing. In the case of 5 Hz sampling, the periodic input to the locusts' photoreceptors would have occurred only every fourth wingbeat, yet this infrequent stimulus was occasionally enough to precipitate entrainment. We managed to record both phase-locked and free-running sequences within our dataset. Phase-locking was beneficial for these experiments in the sense that it allowed for repeated measurements of a single phase of the wing stroke, while free-running flight sequences had the benefit of scanning through different phases of the cycle.

### 2.2. Tomographic particle image velocimetry set-up

**Figure 1.**
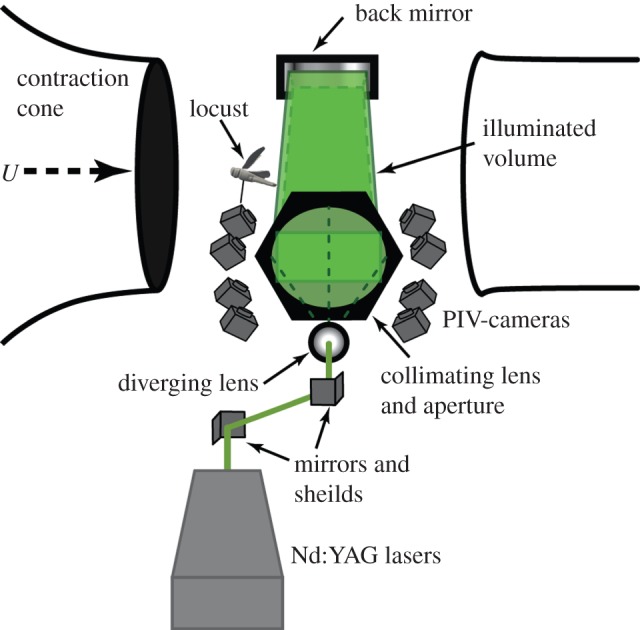
Experimental set-up. An open test section; a closed return wind tunnel was used. The beam from the 1 J pulse laser system was guided via mirrors to a diverging lens followed by a collimating lens and a rectangular aperture which created an illuminated volume that was back-reflected by a large planar mirror on the opposite side of the test section. The locusts were tethered on a sting that allowed for adjustment of body angle and were positioned just in front of the illuminated volume. Eight high-sensitivity cameras captured the volume from below. Photographs of the apparatus are available in the electronic supplementary material, figure S1. (Online version in colour.)

Figure 1 shows a schematic of the apparatus and photographs of it can be found in the electronic supplementary material (figure 1). We used a powerful Nd : YAG laser system comprising two Spectra Physics Quanta Ray Pro laser heads and an external beam combination. Only one of the lasers was frequency doubled to 532 nm to enable a beam combination by a dichroic mirror. The combined beams passed an external crystal for frequency doubling, which only affected the remaining 1064 nm radiation of the second laser head. The laser power of each pulse was around 1 J and the beam profile had a diameter of approximately 10 mm. In order to illuminate the large interrogation volume of about 200 × 240 × 50 mm (streamwise, spanwise, vertical), a spherical *f* = −40 mm lens was used to widen the beam, while a large *f* = 1200 mm lens was used to parallelize the beam again. This way, the beam was widened by a factor of 30, resulting in a diameter of approximately 300 mm. To cut this large beam into the desired illumination shape of the interrogation volume, a rectangular aperture of 200 × 50 mm was installed directly in front of the second lens. Cutting away large parts of the laser beam ensured homogeneous illumination conditions within the measurement volume limited by a sharp intensity decline. In order to increase particle light intensity and to get forward-scattering in all cameras, the light was reflected back with a laser mirror at the opposite side of the test section. To prevent the light from re-entering the laser, the mirror was slightly offset laterally and the returning laser beam was finally deflected into a beam dump.

The air was seeded with di-2-ethylhexyl-sebacate (DEHS) droplets (particle size approx. 1 µm) downstream of the test section. Eight high-sensitivity cameras (LaVision Imager sCMOS, 2560 × 2160 px resolution; LaVision GmbH, Germany) were used to record the flow within the measurement volume. Only the high sensitivity of the sCMOS cameras allowed for recording at a depth of field of 50 mm at the necessary high *f*-number (*f* = 11). In previous experiments with a similar measurement volume 2 × 2 hardware binning had to be used with 11 Mpx cameras to gather enough light [23]. The cameras were equipped with macro lenses (Sigma 105 mm f/2.8 EX DG Macro) and mounted on Scheimpflug adapters (LaVision V3). The cameras were all positioned underneath the test section in a pyramidal 2 × 4 grid formation (streamwise × spanwise). The use of eight cameras allowed for successful tomographic reconstruction on images with high particle density, which is required in order to retain an acceptable spatial resolution within such a large measurement domain. The realized particle density was estimated to be between 0.1 and 0.4 ppp (particles per pixel). Cameras and lasers were synchronized using two programmable timing units (PTU 9; LaVision), connected to two recording PCs controlling four cameras each. Recording, calibration, volume self-calibration, volume reconstruction and volume correlation were done using DaVis 8.1 (LaVision).

### 2.3. Particle image velocimetry camera calibration

The initial perspective camera calibration was performed using a single view of a three-dimensional calibration target (LaVision type 31, 300 × 300 mm^2^) with calibration markers on two levels, separated by 3 mm in the out-of-plane direction. The calibration target was lowered into the centre of the volume from above using a stepper motor-controlled arm which ensured repeatable placement each time. The calibration model used was a third-order polynomial for each plane with linear interpolation of the planes. The residual calibration error for all eight cameras was between 0.10 and 0.15 px. This low value reflects the good quality of the optical set-up (e.g. Scheimpflug adjustment and focus) and can be regarded as a solid base for the following volume self-calibration procedure. The calibration based on the calibration target was repeated several times a day, to compensate for minor dislocations of the camera set-up, originating from temperature changes or mechanical stress during wind tunnel operation.

### 2.4. Volume self-calibration

To achieve the required calibration accuracy for tomographic reconstruction of 0.1 voxels [6], volume self-calibration was applied to each recording sequence [24]. The technique uses the particle images for the correction of unavoidable residual calibration inaccuracies. In this way, the residual calibration errors could be detected and reduced from approximately 0.6 voxels (maximum) to 0.08 voxels (maximum). Although the stability of standard volume self-calibration has been questioned for more than four cameras [25], no problems were observed in this study using eight cameras and the standard volume self-calibration procedure.

### 2.5. Image preprocessing

Standard image preprocessing for tomo-PIV typically includes the subtraction of a local minimum to get rid of the camera background intensity (about 300 counts for these sCMOS cameras). However, at the high particle density (approx. 0.1–0.4 pp) used in the current experiment, the overlapping particles are so close together that often the local minimum is much higher than the camera background. Consequently, removing a local minimum in this situation would remove parts of the true particle intensity. To avoid this, a minimum over time was calculated using images recorded at a low particle density. This minimum image was subtracted from all recordings at higher seeding densities. No further image preprocessing was applied, such as smoothing, since this was found to increase the source density and to boost the reconstruction artefact intensity (often called ‘ghosts' [6]).

### 2.6. Tomographic volume reconstruction

A three-dimensional voxel space containing the particle intensity information was reconstructed from the recorded camera images using MLOS-MART (multiplicative line of sight—multiplicative algebraic reconstruction technique [6,26]; with eight iterations and intermediate volume smoothing [25]). The reconstructed volume had an extent of 200 × 240 × 58 mm^3^, corresponding to 2250 × 2700 × 650 voxel with 0.089 mm/voxel.

### 2.7. Velocity field calculation

From the reconstructed three-dimensional particle distributions at two subsequent time instants, the velocity fields were calculated using fast direct three-dimensional correlation with a multi-pass approach and decreasing interrogation volume size [27]. The volume used for correlation was smaller than the reconstructed volume (2250 × 1900 × 580 voxels), removing borders with lower reconstruction quality in the out-of-plane direction and concentrating on the region of interest in the spanwise direction. The final effective interrogation volume size applied was 48 × 48 × 48 voxel with 75% overlap and Gaussian weighting. This results in a vector spacing of 1.07 mm and a total number of 187 × 160 × 48 velocity vectors for each instantaneous measurement.

## 3. Analysis

### 3.1. Flow patterns and their consistency

The PIV volume was large enough to capture instantaneously a whole wavelength and the complete amplitude for most of the individuals, although at some instances the lowest part of the downstroke was just outside of the volume. Vortex patterns were examined visually to identify recurring structures in the wake. With the unprecedented information offered by the large volume and high resolution of the measurements, many smaller structures were revealed that have not been described before. We sought to ascertain which of these features are periodically consistent structures generated by the animals, which of them are more occasional features and which are merely artefacts of noise. Individual frames (representing single instantaneous measurements) give the highest detail of the small, intricate structures but can be more difficult to interpret due to measurement noise. A powerful method of removing non-periodic elements of the wake is to use proper orthogonal decomposition (POD). With the ‘method of snapshots’ by Sirovich [28], the vector field is decomposed into orthogonal temporal and spatial modes, assuming that the vector field *U*(*x*, *t*) can be approximated as follows:
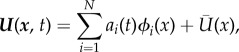
where *a*_i_ and *ϕ_i_* are temporal and spatial modes, respectively, and *U*(*x*) is the mean vector field. Note that the modes are sorted in descending order by the energy in each mode.

**Figure 2.**
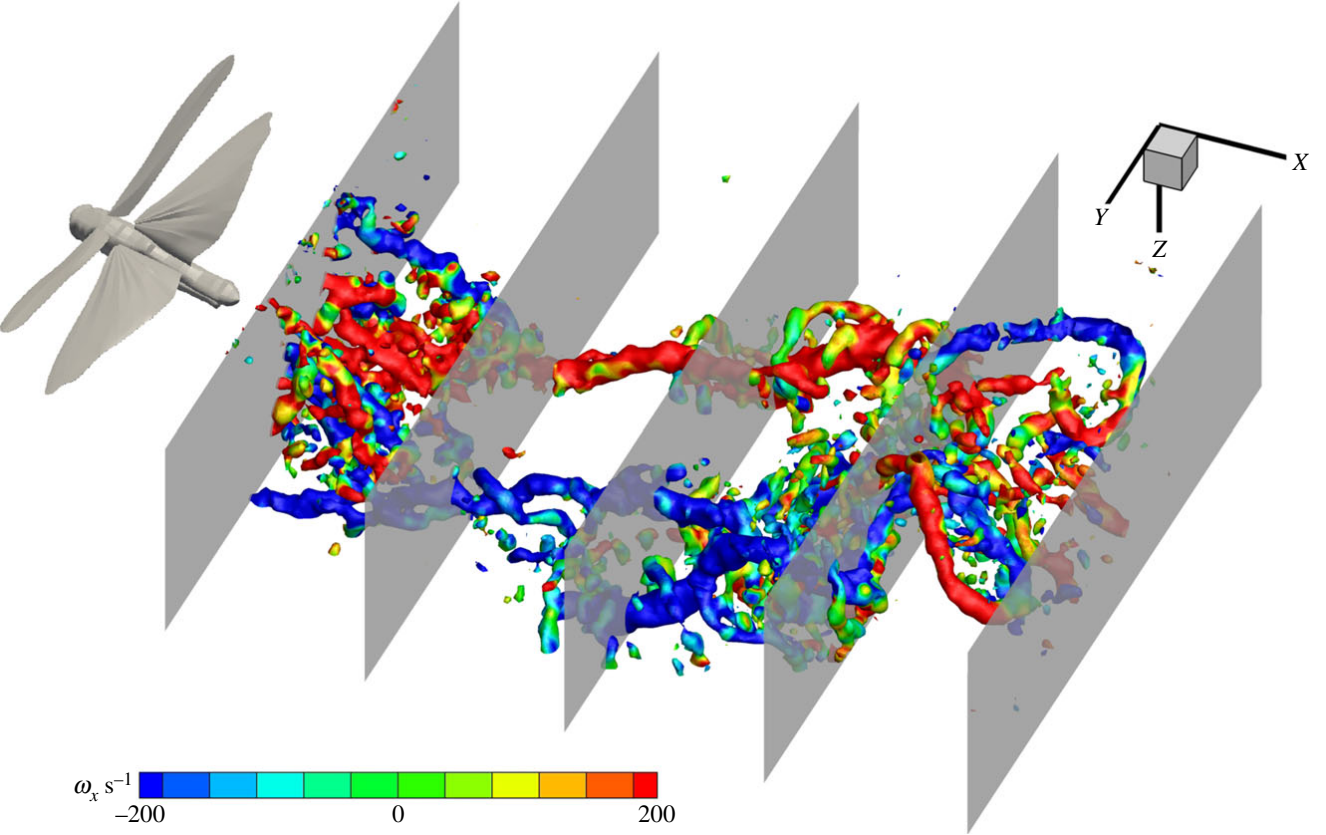
The position of the five extracted planes from the volume used for examining wake development and deformation. The wake is presented as *Q*-criterion iso-surfaces at *Q* = 0.01 coloured by *x*-vorticity.

This technique is highly suited to the type of data gathered in these tethered flight experiments because the animal is held in place, meaning that the wake is spatially consistent. Here, we used POD as a means to determine the consistency of various features of the wake. Because measurement noise is non-periodic, POD processing also had the effect of cleaning the plots and easing the task of interpretation. The fewer modes that are used for POD reconstruction, the more elements disappear. We exploited this effect by examining the wake features of one sequence (non-phase-locked) in detail using 6, 10, 23, 56, 105 and 230 modes (out of 230 in total), corresponding to 70%, 75%, 80%, 85%, 90% and 100% of the total energy in the wake. The relative energy distribution in the POD modes and the cumulative sum of energy is shown in the electronic supplementary material (figure 2).

### 3.2. Force and span efficiency estimates from transverse planes

**Figure 3.**
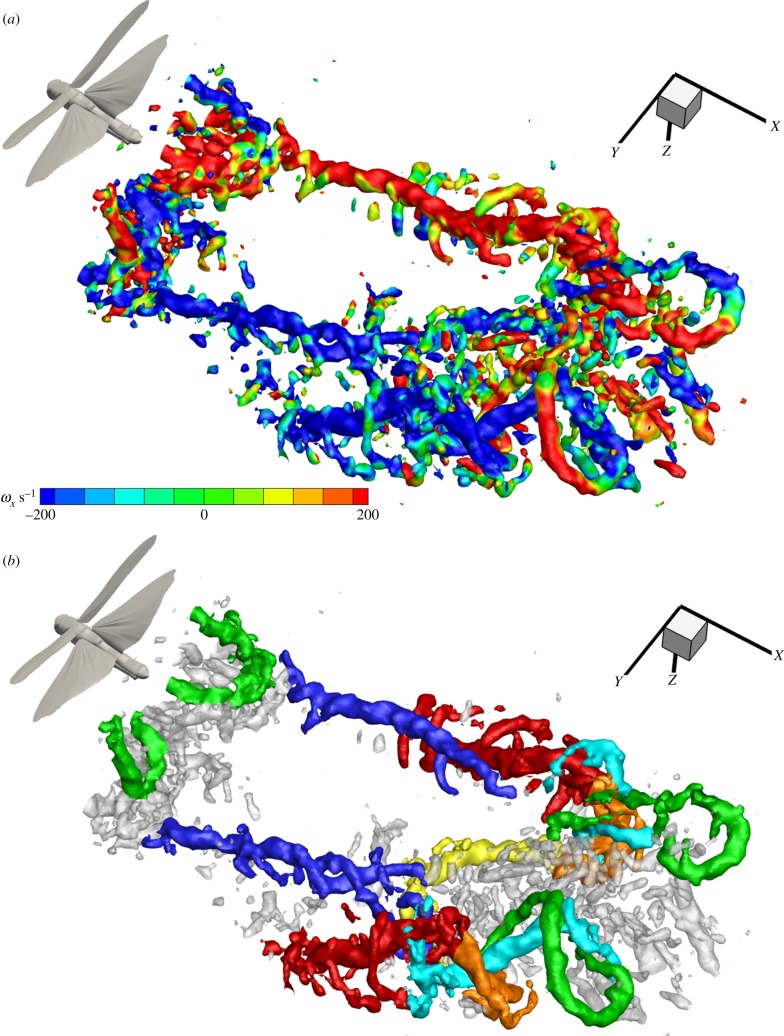
Instantaneous representation of the full wingbeat wavelength. (*a*) *Q*-criterion iso-surfaces at *Q* = 0.01 coloured by *x*-vorticity. (*b*) Wake elements colour coded by origin; green, forewing upstroke tip vortex; cyan, forewing starting vortex and early tip vortex; dark blue, forewing downstroke tip vortex; orange, hindwing downstroke starting vortex; yellow, hindwing downstroke root vortex; red, hindwing downstroke tip vortex. An interactive three-dimensional PDF version of (*b*) is available in the electronic supplementary material, figure S4.

A common goal of animal flight wake studies is to calculate the aerodynamic efficiency of flight (e.g. [10,29]); yet, there is little consistency in the position of the stereo-PIV measurement plane relative to the animal and this downstream distance is likely to affect the data if the wake is undergoing significant deformation as it convects away from the animal [8,14]. In order to investigate how lift and span efficiency estimates might be affected by how far downstream the data are recorded, we extracted transverse planes from five locations within our instantaneous volumes; the first plane was closest to the animal (just behind the trailing edge), the last plane was furthest away and three more were evenly spaced between these (figure 2). Thus, from the five sequences corresponding to the five individuals, 25 new sequences each with 230 frames of planar PIV vector fields (five for each individual) were generated to mimic stereo-PIV planes at increasing measurement distances downstream of the animal. Each sequence contained various wingbeat phases—all recorded from different wingbeats due to the sampling rate being lower than the wingbeat frequency. Therefore, each sequence and frame needed to be sorted by phase to build a pseudo-time-resolved sequence. This was done by using the first (dominant) mode of the POD reconstruction under the assumption that this mode consistently assigns the same phase to frames captured at the same phase of the wingbeat. The first mode is always dominated by the wingbeat frequency so it was trivial to convert the phase of each frame from POD phase to wingbeat phase. POD in this case was only used to find the phase of each frame and not to make POD approximations—the frames used for phase averaging were raw vector fields (see the electronic supplementary material (figure 3) for details on phase averaging). This simple method proved very effective. After each frame was sorted by phase, they were binned with respect to time to generate a phase-averaged wingbeat containing 48 frames, the equivalent of time-resolved data captured at *ca* 1 kHz. Following the method described by Henningsson & Bomphrey [5], the phase-averaged frames were analysed by manually pinpointing the centres of the wing-tip vortex of the hindwing in each frame and a velocity transect extracted between these and a common rotational centre. Lift was then calculated as
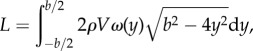
where *y* is the spanwise location, *ρ* is the density of the air, *V* is the freestream velocity, *ω*(*y*) is the spanwise vertical velocity and *b* is the span of the wake. Span efficiency was calculated as
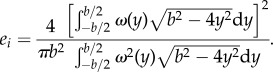
The method for calculating span efficiency and lift is described and explained in detail in [5], so the reader is referred to this article for further information. Velocity transects from the centre of the body to the wing-tip vortices, including the upwash outboard of their axes, were used to calculate lift force and span efficiency.

### 3.3. Estimates of wake convection and deformation

Instead of using velocity distribution transects behind the trailing edge of the wings, it is also possible to estimate lift and thrust forces using a simplified vortex ring model. In this case, the geometry of the wake is scrutinized to extract the diameter of the vortex cores, the diameter of the vortex ring (often taken to be the wing span), and the plane of the ring (either in its entirety, or section by section). In the previous tomo-PIV study of locust flight, the collection of mini-volumes was rearranged to hypothesize how much the wake angle (the plane of an idealized vortex ring, based on the hindwing tip vortex angle) might be expected to change as the wake convects downstream [8]. Here we can measure this directly to test that estimate. We measured the tip vortex angle using a custom Matlab script (The MathWorks, Inc., Natick, MA, USA) to draw a line through the axis of the hindwing's downstroke tip vortex and measure the angle it makes with the horizontal plane. This gives a good indication of the change in the gross wake angle since the hindwing downstroke wake is the strongest feature of the wake, although we note that other parts of the complex vortex wake will convect in different directions. We calculated wake angles using phase-averaged extracts of mid-wing longitudinal planes for five sequences (of different individuals) that showed the wake angles clearly.

## 4. Results

### 4.1. Flow patterns and their consistency

The wake of the locust is a complex arrangement of vortex structures (figure 3). The increased detail of the measurements granted by large-volume tomo-PIV shows many small, delicate structures that have not been visible before. The wake is consistent with previous reports [8], dominated by tip vortices trailing behind both wing pairs during the downstoke that indicate strong downwash and high loading (figure 3*a*). The beginning of the downstroke shows transverse vortex elements formed behind the hindwings as a result of increasing circulation around the wings (figure 3*b*, orange). These elements link the hindwing-tip vortices (figure 3*b*, red) to the hindwing root vortices (figure 3*b*, yellow) which are present during the first half of the downstroke. The root vortices then inflect inwards towards the plane of symmetry and connect in the wake behind the body. The forewing starting vortex and early downstroke tip vortex form a rather complicated three-dimensional shape (figure 3*b*, cyan) with continuation into more stereotypical trailing wing-tip vortices (figure 3*b*, dark blue) similar to those shed by the hindwings. During the upstroke, the forewings have a negative airspeed which results in arching trailing vortices tracing the path of the wing tips first backwards, then up and forwards (figure 3*b*, green). The opposite sense of rotation highlights momentary negative loading [8]. An interactive three-dimensional PDF version of figure 3*b* is available in the electronic supplementary material, figure S4.

**Figure 4.**
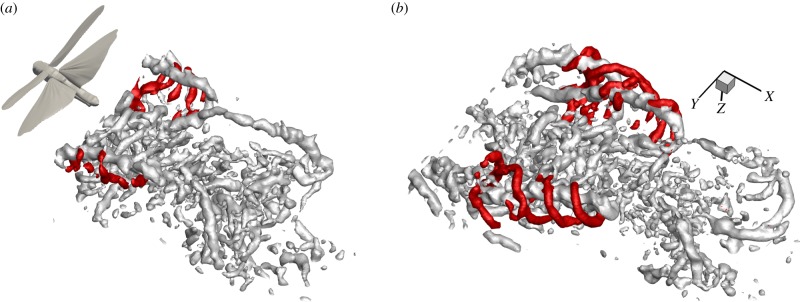
The formation of helical structures outside the wing-tip vortices of the hindwing from a non-phase-locked sequence. (*a*) The transverse vortex structures, highlighted in red, as they roll up behind the wings as a result from Kelvin–Helmholtz instabilities in the shear layer. (*b*) Shortly after formation the vortices get entrained around the strong hindwing downstroke vortices and form helical structures around these.

Alongside these anticipated features we can also see additional, undescribed, wake elements. During the downstroke, distinct vortex structures become apparent that are oriented in the spanwise direction (figure 4).

This roll up is representative of Kelvin–Helmholtz instability in the shear layer behind the trailing edges of the wings. As reported with respect to dragonflies using smoke streak visualizations [30] and inferred behind hawkmoths from two-dimensional PIV [31], the shear layer rolls up into a one-sided wake immediately behind the wing, becoming a series of transverse vortices with the same sense of rotation. Here, we see that they extend across the span of each wing (figure 4*a*). These data also reveal how the Kelvin–Helmholtz vortices are rapidly stretched and entrained around the strong wing-tip vortices forming helices that spiral outside the core of the wing-tip vortex (figure 4*b*). Figure 4*a,b* shows two consecutive frames from the same sequence. They are separated by 0.1 s and therefore represent two different wingbeats yet the flow field is, typically, very consistent. The phase shift is approximately one-third of the stroke cycle.

**Figure 5.**
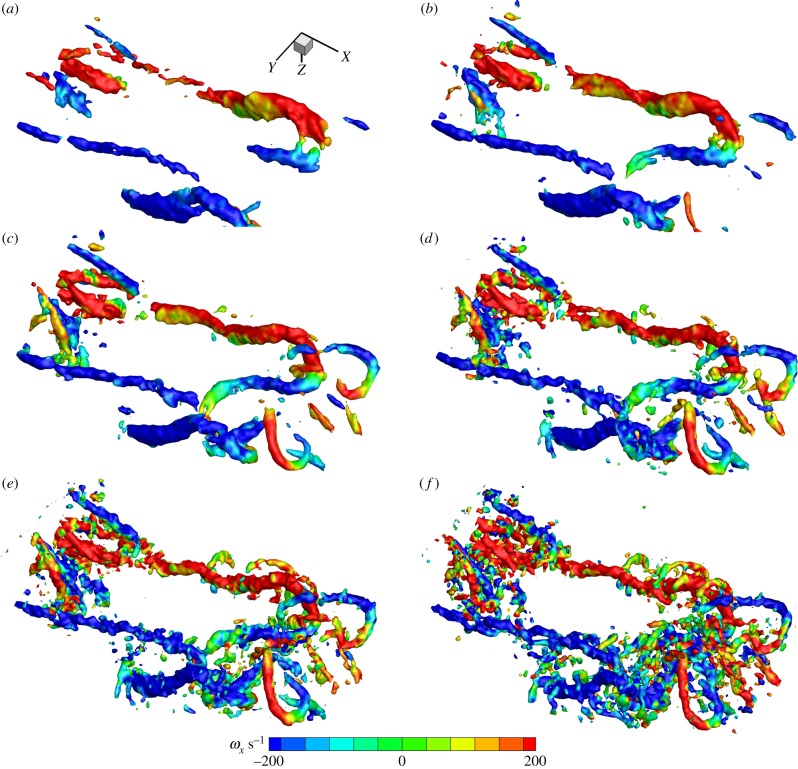
Comparison of consistencies of different structures in the wake by the use of POD reconstruction using different numbers of modes. (*a*) Six modes—downstroke tip vortices of both wing pairs are visible and the root vortex of the hind wing; (*b*) 10 modes—upstroke arches of forewings appear; (*c*) 23 modes—hindwing root vortices link up centrally; (*d*) 56 modes—first sign of entrained Kelvin–Helmholtz vortices; (*e*) 105 modes—Kelvin–Helmholtz vortices clearly visible; (*f*) 230 modes (no POD)—many small structures appear that are not visible in the lower mode reconstructions.

To investigate which structures were most consistent across multiple wing strokes, we performed POD on one of our sequences (non-phase-averaged) using a variable number of modes for the reconstruction. This routine was effective in discriminating between the periodic and non-periodic features of the wake (figure 5). As expected, the most highly periodic structures are created during the downstroke of the forewings and the hindwings, with tip, root and starting vortices consistently present, even with only six modes used for the flow-field reconstruction; six modes corresponds to just 70% of the energy in the wake (figure 5*a*). When 10 modes are used (figure 5*b*), part of the upstroke arches are also visible, and at 23 modes they are almost fully represented (figure 5*c*). At 23 modes, we can also see that the two root vortices from the hindwings are linked behind the body and we start to see the first indications of the entrained Kelvin–Helmholtz vortices (figure 5*c*). These are more apparent at 56 modes (figure 5*d*) and at 105 modes they appear clearly (figure 5*e*). Finally, at 230 modes—which represent the full fidelity data—we see many smaller features that do not appear in the reduced POD reconstructions, suggesting that they are non-periodic and likely to be of little significance to the locust in steady flight (figure 5*f*). Conversely, many of the smaller structures that, at first glance, may have appeared to be the result of measurement noise, especially those behind the body, are still retained at 105 modes. This suggests that they are likely to be genuine, albeit not as spatially and temporally consistent as the principal structures. Further examination of the Kelvin–Helmholtz vortices and the POD modes that correspond to them is available in the electronic supplementary material, figure S5.

### 4.2. Force and span efficiency estimates from the transverse plane

**Figure 6.**
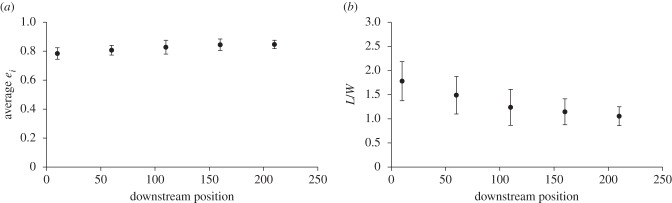
(*a*) Span efficiency at the five different downstream locations. There is no significant change over the measured distance. (*b*) Weight support at the five different downstream locations. Weight support decreases with downstream location, but appears to level out towards the downstream end.

We calculated aerodynamic span efficiency for each of the five locusts and simulated stereo sampling planes from five downstream locations using our volumetric data. The mean span efficiency of the five locusts and samples from all five downstream locations was *e_i_* = 0.82, higher than the figure of 0.53 reported previously [5]. Span efficiency did not change significantly with downstream distance of the vector fields (ANOVA; *p* = 0.087). The values stay close to constant regardless of whether the vector field that was used for the estimate was extracted from the plane closest to the animal or the plane furthest away (figure 6*a*). This is an encouraging result in the context of the robustness of flight efficiency studies using PIV.

By contrast, the measured weight support (calculated as lift/weight) decreases as the measurement plane moves further downstream (ANOVA; *p* = 0.018). The variation is fairly large across the different individuals flown in the tunnel, but they were all producing more lift than necessary to support their own weight. The decline in calculated lift does not appear to be linear but, instead, seems to asymptotically approach a stable lower level within the distance covered by the measurement volume (figure 6*b*).

**Figure 7.**
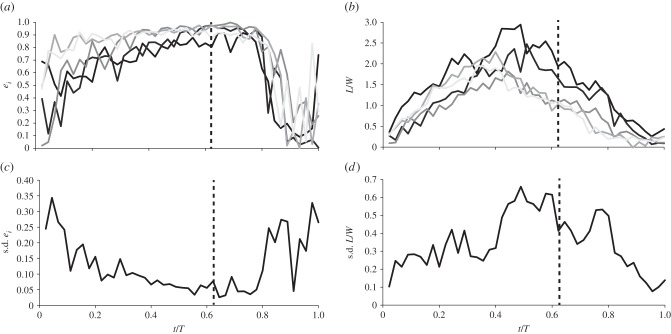
(*a*) Dynamics of span efficiency throughout the wing stroke at the five different downstream locations. (*b*) Weight support throughout the wing stroke at the five different downstream locations. Darker shaded lines indicate shorter downstream distance between the animal and the putative measurement plane; from 10 mm closest to the locust to 210 mm, in increments of 50 mm. (*c*) Standard deviation of span efficiency between the five downstream locations. (*d*) Standard deviation of weight support.

To examine this further, we plotted *e_i_* and *L*/*W* over the wingbeat for each of the five downstream locations of one of the individuals. Standard deviation of the parameters at each time step was used to illustrate where in the wingbeat the biggest discrepancy between locations occurs (figure 7). It is clear from this figure that *e_i_* has the lowest discrepancy during the period in the wing stroke where most of the lift is produced, whereas *L*/*W* shows the opposite trend; around mid-downstroke when the peak of lift occurs the discrepancy between measurement locations close and far is also the highest.

Across the five different downstream locations, the average wake span over the wingbeat changed from 64 to 55 mm. This represents wake contraction to approximately 86%.

### 4.3. Estimates of wake convection and deformation

**Figure 8.**
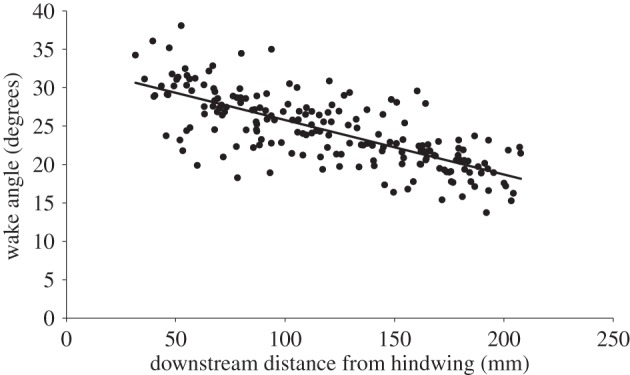
Wake angle over downstream distance. As the wake convects downstream, the angle decreases resulting in a more horizontal wake. Regression: *y* = −0.071*x* + 32.9, *R*^2^ = 0.54.

In the time it takes for the wake to pass through the length of the measurement volume (which is approx. one wavelength of the stroke cycle), the wake evolves and deforms to a large degree. One of the clearest changes was the vertical convection of the wake due to the induced downwash. The portion shed at the beginning of the downstroke convects downwards at approximately the same velocity as the wing tip, which leads to the overall wake angle of the downstroke becoming more horizontal. The wake angle changed from about 31° to 18° over the distance of the measurement volume (figure 8), which, if viewed in isolation as a volumetric snapshot, can give a misleading impression of the fluid dynamics relevant to the animal [8].

## 5. Discussion

### 5.1. Flow patterns and their consistency

This set of experiments comprises the most detailed depiction of the wake of any flying animal to date. It is the first study where a complete wing stroke could be captured in a single instantaneous measurement with three-dimensional representations of velocities inside the volume. We find that the wake of the locust is more complex than described previously. The aerodynamics of locusts have been extensively studied in animal flight experiments over a number of decades with increasingly advanced fluid diagnostic tools, each revealing more intricate detail of the wake. In addition to the structures revealed by small-volume time-resolved tomo-PIV [8], we find that the wake consists of several small, yet consistent, structures that have not been described before. One of the most conspicuous features is the series of transverse vortices that roll up immediately behind the trailing edge of the wings as a consequence of Kelvin–Helmholtz instabilities in the shear layer. This instability has been inferred previously in animal flight [30,31] and here we present confirmation of its presence and detail of its structure. We expect this to be a common feature of non-impulsively started wings where a shear layer is generated during the initial acceleration phase following pronation.

Entrainment of the Kelvin–Helmholtz vortices around the trailing vortices results in eye-catching helical structures spiralling outside of the wing-tip vortex. The fluid behaviour is highly consistent and appears in all sequences. Over the distance covered by the measurement volume these vortices do not merge into the wing-tip vortex but remain as discrete secondary structures. In the more developed wake, these structures might be mistaken for secondary elements (or an imperfect representation) of the wing-tip vortex but they should not be considered as such.

Although this phenomenon has not been described for flying animals, there is a recent description of these smaller vortices orbiting the stronger wing-tip vortices trailing behind rotating mechanical wings [32]. Mechanical flappers confer the advantage of volume reconstruction from multiple measurement locations along the wing span and repeatable wing strokes, where animal behaviour and kinematic variations become irrelevant. Helical structures around wing-tip vortices have also been found in computational fluid dynamic modelling of butterfly flight [33], but it was not clear where these structures originate from, only that they appear close to the tip of the wing. The consistency of the structures is high as they appear in more or less every instantaneous measurement and they are excellent examples of structures that are only captured behind live animals using volumetric techniques. The challenge arises because both the cores are small and they are often oriented with their axes in the transverse plane, making them difficult to detect even when using time-resolved, transverse PIV (cf. [8,9]). It is likely that this type of structure has been partially captured in previous transverse measurements but, owing to limitations in between-plane spatio-temporal resolution, they may have been dismissed as inconsistency in the vector fields and assumed to be part of the wing-tip vortex. Thus, we warn against excessive smoothing of PIV data. POD using a variable number of modes showed the periodicity of smaller structures, such as the Kelvin–Helmholtz vortices and the smaller lateral vortices connecting the wakes of the left and right wings. This emphasizes the importance of accurate three-dimensional reconstructions because smaller sub-structures can be put into context, revealing the genuine wake geometry.

### 5.2. Force and span efficiency estimates

Our analysis of how estimates of span efficiency and weight support vary depending on how far downstream measurements are taken showed that span efficiency was more or less unaffected while estimated weight support decreased with distance. As can be seen in figure 7, the discrepancy in span efficiency estimates occurs mainly at the beginning and end of the wing stroke, which is also when the smallest forces are generated and therefore the impact on the mean span efficiency is low. This results in very little change in the span efficiency estimate across the different distances. Lift on the other hand is clearly decreasing with increasing distance downstream of the animal. The wake span decreased over the distance from 64 to 55 mm (86%). The wake of ideally loaded gliding wings contracts to *π*/4 of the original wake span (i.e. 79%), so this suggests that the wake either has not fully contracted within our measurement volume (but remains in an intermediate contraction phase) or other mechanisms are in play. In reality, we know that the loading is not ideal because span efficiency is lower than one, so the full wake contraction is expected to be even more than 79%. Following Bernoulli, we would expect velocities to increase as the wake span decreases but we find, here, that the global mean of velocities, across the span and over the wingbeat, decreases from 0.35 to 0.27 m s^−1^. This may be due to dissipation and explains why the weight support is decreased when estimated from planes that have been measured further downstream in the wake. As shown by Horstmann *et al*. [14], we may also expect the root vortices that are present during parts of the downstroke to interfere with the wing-tip vortices, causing them to pivot downwards. The resultant wake rotation can lead to lower vertical velocity components and, consequently, a lower estimate of lift force. This may also explain why the discrepancy in lift is highest around the time of peak lift production since this is also when the strongest root vortices are present and the likelihood of wake rotation is highest.

### 5.3. Wake deformation

After the wake is shed, it continues to develop and deform and one of the clearest changes is the overall downstroke wake angle. The angle of the downstroke wake as measured from a plane captured close to the animal (corresponding to the upstream part of our volume) is steeper than if the same angle were to be measured from a wake plane captured far from the animal (corresponding to the downstream end of our wake volume). This type of deformation or development had been predicted recently based on the results of time-resolved, small-volume tomo-PIV [8]. The results confirm the qualitative prediction and closely match the quantitative prediction. The wake angles prior to deformation and post deformation were both consistent to a precision of 1°. The downstroke wake angle, here, is 31°, calculated using a fitted regression at a distance of 10 mm behind the hindwing trailing edge. The initial wake angle in our earlier study was 32° [8] and was predicted to relax to 20° within one wavelength of convection. Here, one wavelength behind the animal, at the furthest measureable distance downstream of 210 mm, the regression analysis yields a downstroke wake angle of 18°, confirming the earlier prediction (figure 8).

Wake angle changes present significant challenges when using downstream measurements, or low temporal resolution, large-volume measurements for estimating the lift and thrust acting on an animal, because the average resultant aerodynamic force vector also changes (in this case closer to vertical). This modulation can give the misleading impression of higher lift and lower thrust than was actually generated by the wings. Simply considering the change in the angle found here over the distance of the measurement volume (13°) would result in an overestimate of lift of 110% and an underestimate of thrust of 60%.

### 5.4. The benefits and limitations of large-volume tomographic particle image velocimetry

Although the resulting wake reconstructions from these experiments are exceptionally detailed, there are some practical limitations to the large-volume tomo-PIV method. A powerful laser system was used for these experiments alongside eight high-resolution, high-sensitivity cameras. In addition to this study, the tomo-PIV system was used within the scope of the PIV course (http://pivcourse.dlr.de) at the German Aerospace Centre (DLR) in Göttingen. We acknowledge that the cost of this equipment means that it will not be practical to replicate this set-up in many laboratories worldwide at this stage. A significant issue for this work was also the extensive processing time required to progress from raw images to finished vector fields. Analysing the dataset for this study took 96 days of around-the-clock processing on a server with 48 AMD cores and 64 GB of RAM. Despite these challenges, it is possible that this technique will become a standard way of measuring aerodynamic tracks in the future, even around much larger swimming and flying animals. High-speed (1 kHz) large-volume (200 × 200 × 100 mm³) tomo-PIV using helium-filled soap bubbles has been demonstrated recently [34] and has even been expanded to 500 × 300 × 200 mm^3^ (F Scarano, S Ghaemi, G Caridi, J Bosbach, U Dierksheide 2015, personal communication). These bubbles may be too large for insect flight wake measurements (300–500 µm) but may prove useful for bird measurements.

When synthesizing our experiences from large-volume, low repetition rate tomo-PIV with those from small-volume, high repetition rate tomo-PIV [8], we conclude that, if resources are limited, a high repetition rate should be prioritized over a larger volume for quantitative analysis of animal flight dynamics. These parameters are often subject to a trade-off due to limitations in laser power and the conflict of frame rate versus pixel resolution in high-speed cameras. High repetition rate, in combination with a small volume, is potentially the most useful, so long as the volume is large enough for reliable estimates of out-of-plane vorticity and to overlap with each other if using a wind or water tunnel. The fact that this large-volume PIV captures the whole wake of the animal is in a way both its benefit and its drawback. By capturing the complete wake instantaneously, the wake we present is a true representation of the wake left behind locusts flying tethered in a wind tunnel and is more accurate than has been possible using composite reconstructions of planar PIV images. However, in terms of aerodynamic analysis, such as inferring forces acting on the animal, this is best done using the vector fields closest to the animal that have had little time to deform. While the additional information added by capturing the ‘older’ parts of the wake further downstream is less relevant in the context of the animal's aerodynamics, tomo-PIV still has the significant advantage over planar PIV that flow derivatives in any axis can be calculated from adjacent vectors that were captured simultaneously. If the aerodynamic forces acting on the animal are not the primary concern, then the full wake pattern is of particular importance. Such situations include investigations of interactive behaviour, for example flocking flight in birds [35,36], swarming in insects [18,37], the schooling of fish [38], predator–prey dynamics [39] or the control of multiple unmanned air systems.

## Supplementary Material

Supplementary figure 1

## Supplementary Material

Supplementary figure 2

## Supplementary Material

Supplementary figure 3

## Supplementary Material

Supplementary figure 4

## Supplementary Material

Supplementary figure 5

## Data accessibility

The dataset has been uploaded to the Dryad Digital Repository: http://dx.doi.org/10.5061/dryad.1cn55.
