# Supplementary material for: The complex aerodynamic footprint of desert locusts revealed by large-volume tomographic particle image velocimetry
Source: J R Soc Interface. 2015 Jul 6;12(108):20150119. doi: 10.1098/rsif.2015.0119 (PMC4528577; doi:10.1098/rsif.2015.0119)
Supplement: Supplementary figure 1 [file rsif20150119supp1.pdf]

Supplementary figure 1

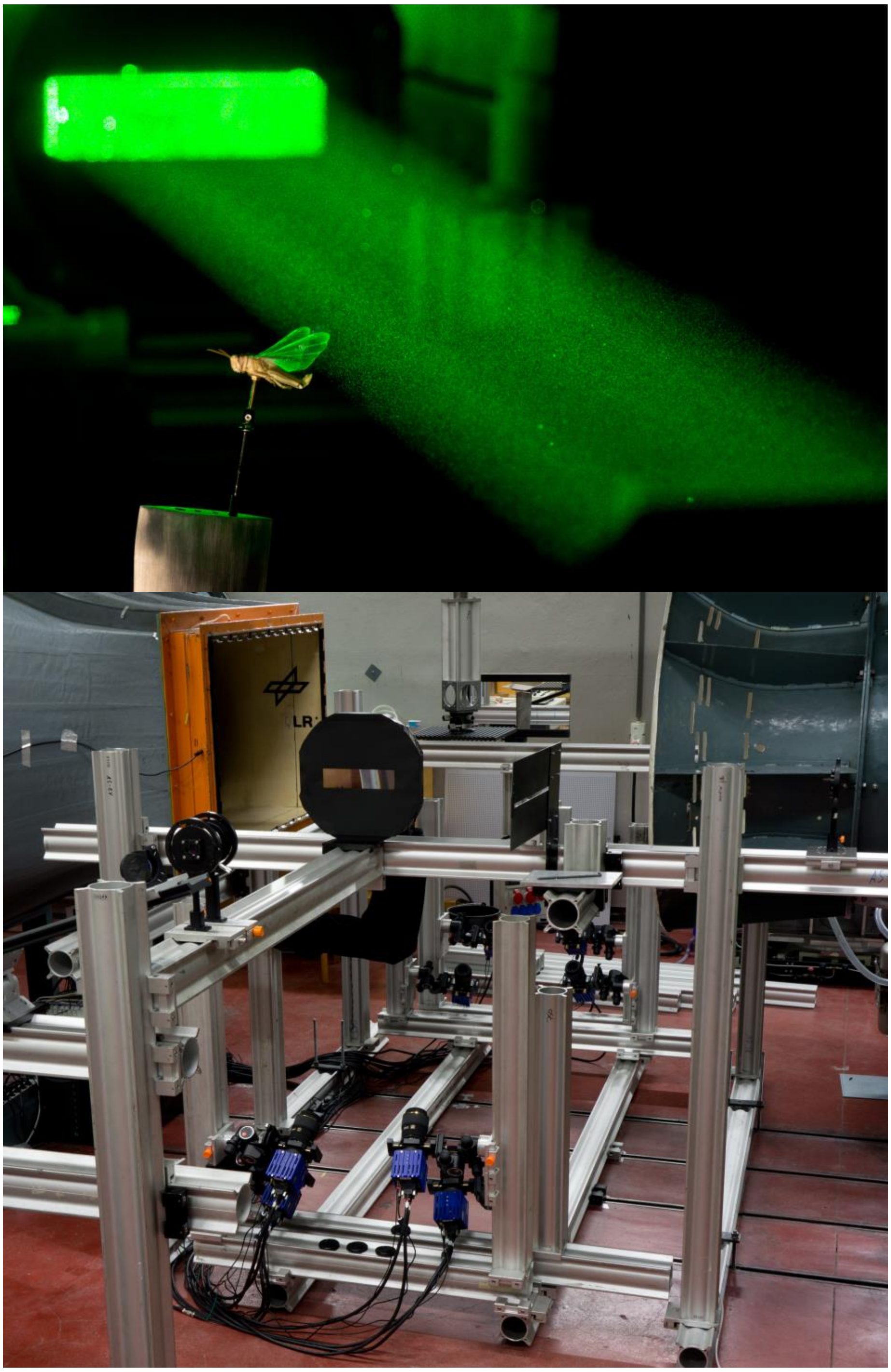

Top panel: The locust in flight on the tether in front of the illuminated volume. Lower panel: The PIV apparatus. In this photograph the calibration plate is lowered into the measurement volume. Photos: Daniel Schantz
