# Supplementary material for: The complex aerodynamic footprint of desert locusts revealed by large-volume tomographic particle image velocimetry
Source: J R Soc Interface. 2015 Jul 6;12(108):20150119. doi: 10.1098/rsif.2015.0119 (PMC4528577; doi:10.1098/rsif.2015.0119)
Supplement: Supplementary figure 2 [file rsif20150119supp2.pdf]

Supplementary figure 2

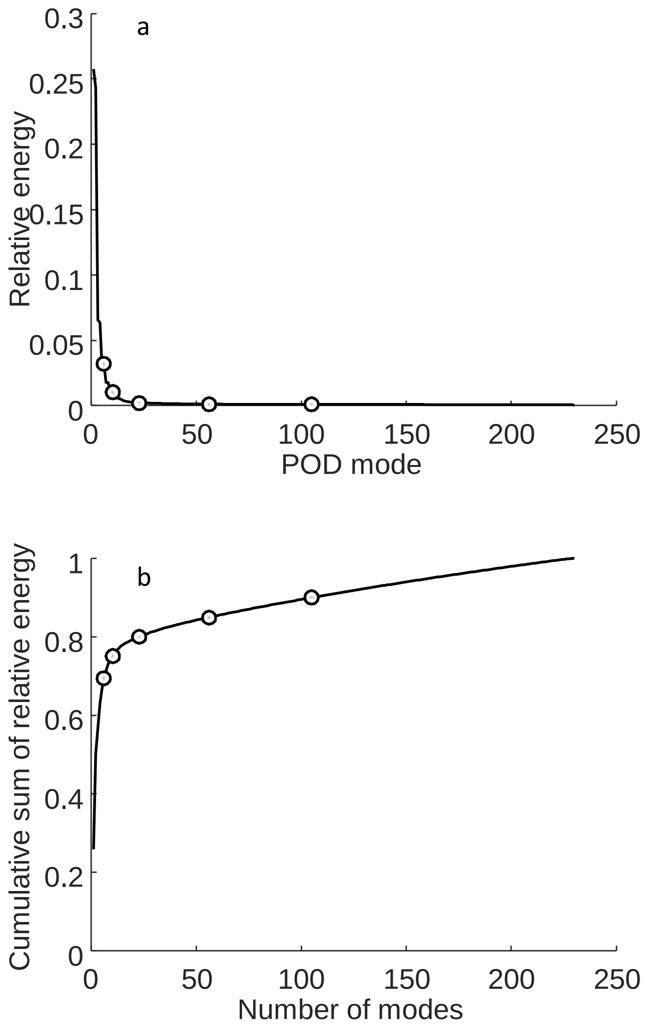

Relative energy distribution in POD modes. The mode number (5, 10, 23, 56 and 105) used for the comparison of consistencies in figure 5 are shown by circles. a) Relative energy in each POD mode scaled so as to sum to 1. b) Cumulative sum of the relative energy in POD mode.
