# Supplementary material for: The complex aerodynamic footprint of desert locusts revealed by large-volume tomographic particle image velocimetry
Source: J R Soc Interface. 2015 Jul 6;12(108):20150119. doi: 10.1098/rsif.2015.0119 (PMC4528577; doi:10.1098/rsif.2015.0119)
Supplement: Supplementary figure 3 [file rsif20150119supp3.pdf]

Supplementary figure 3

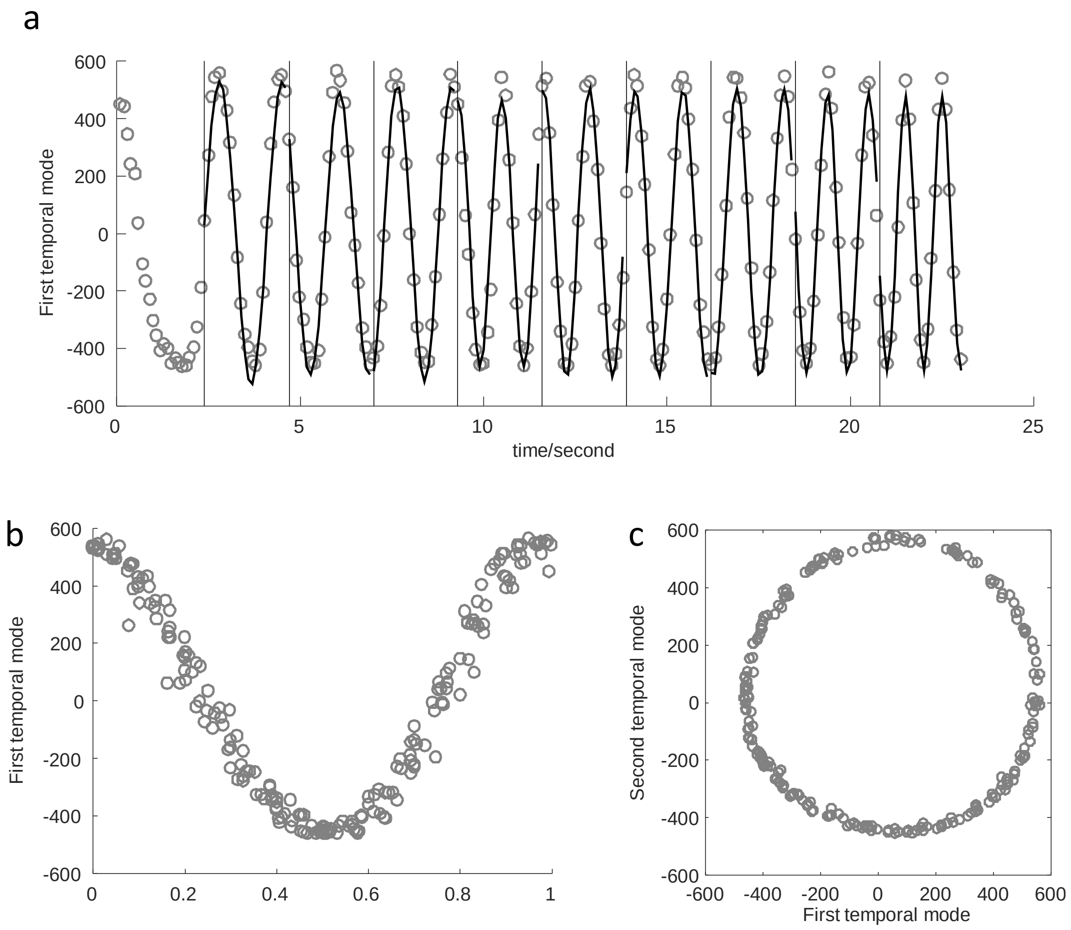

Reconstruction of phase-averaged wingbeat with fitted sinusoidal curves. a) The first temporal mode (circles) and sinusoidal curves fitted by Levenberg-Marquardt algorithm in each window. The first window in this sequence is neglected due to phase-lock. b) The temporal modes arranged by the phase derived by fitted curves. c) First and second temporal modes.
