# Supplementary material for: The complex aerodynamic footprint of desert locusts revealed by large-volume tomographic particle image velocimetry
Source: J R Soc Interface. 2015 Jul 6;12(108):20150119. doi: 10.1098/rsif.2015.0119 (PMC4528577; doi:10.1098/rsif.2015.0119)
Supplement: Supplementary figure 4 [file rsif20150119supp4.pdf]

Supplementary figure 4

(Instantaneous representation of the full wingbeat wave length)

Instantaneous representation of the full wingbeat wave length. Wake elements colour coded by origin; green: forewing upstroke tip vortex, cyan: forewing starting vortex and early tip vortex, dark blue: forewing downstroke tip vortex, orange: hindwing downstroke starting vortex, yellow: hindwing downstroke root vortex, red: hindwing downstroke tip vortex. To maintain a reasonable file size, the unlabelled (grey) features have been downsampled to 20 % of the original facets.
