# Supplementary material for: The complex aerodynamic footprint of desert locusts revealed by large-volume tomographic particle image velocimetry
Source: J R Soc Interface. 2015 Jul 6;12(108):20150119. doi: 10.1098/rsif.2015.0119 (PMC4528577; doi:10.1098/rsif.2015.0119)
Supplement: Supplementary figure 5 [file rsif20150119supp5.pdf]

Supplementary figure 5

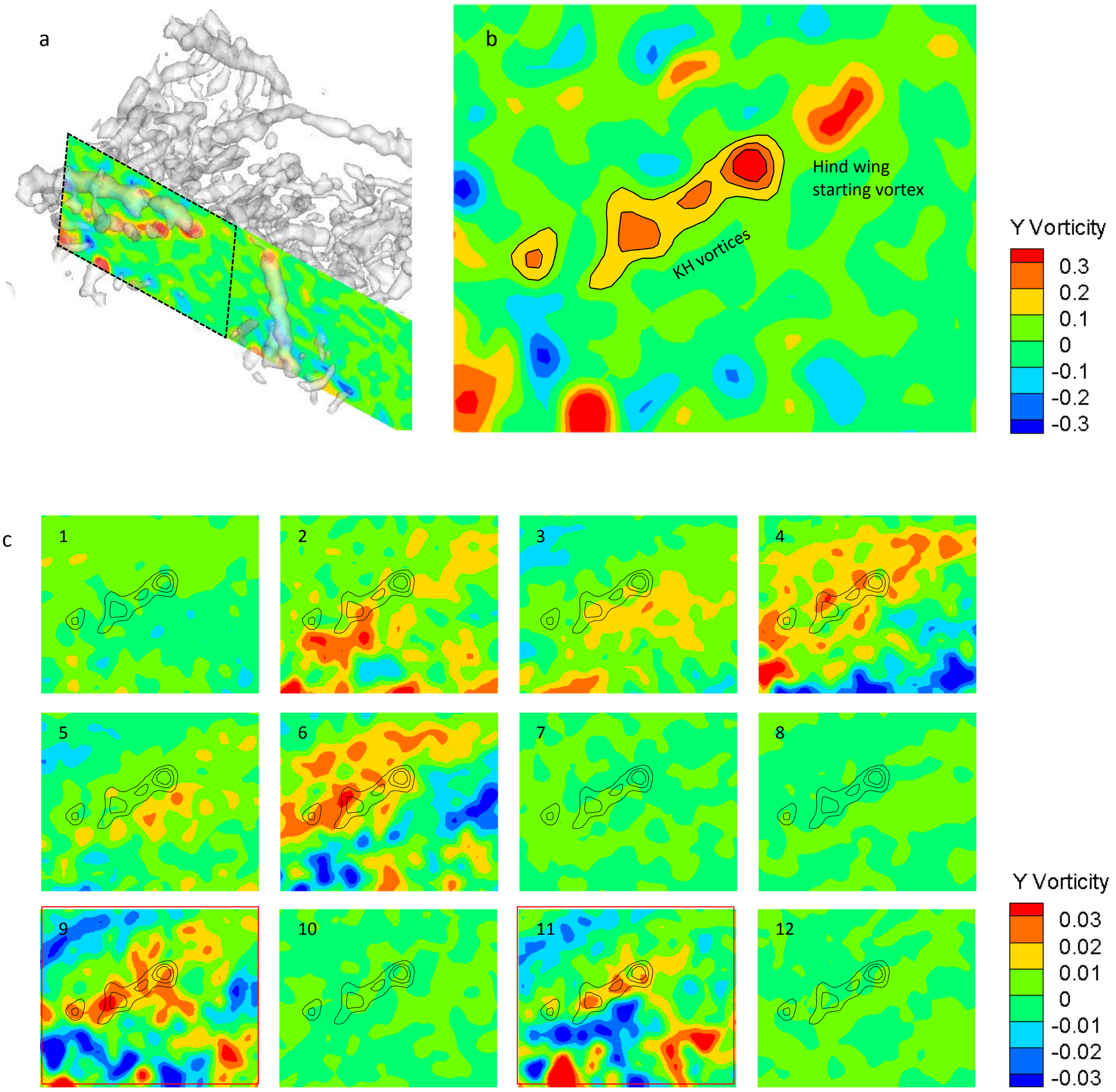

Transverse vortex structures as a result from Kelvin-Helmholz instabilities and the spatial modes of POD at same time frame. a) Iso-Q surface and longitudinal slice with y-vorticity contour (see figure 4a for complete structures). b) Close-up of the slice in a). The vortices due to the KH instabilities are highlighted by black lines. c) y- vorticity contours of POD spatial modes weighted by corresponding temporal mode. The mode number is shown at the top-left of each panel. The vorticity contour by KH vortices in a) (black lines) are superimposed. The position of strong positive vortices on 9th and 11th modes nicely match with the KH vortices, suggesting that those modes represent a large part of the KH vortices in this sequence.
